# Differential Morpho-Physiological and Biochemical Responses of Duckweed Clones from Saudi Arabia to Salinity

**DOI:** 10.3390/plants12183206

**Published:** 2023-09-08

**Authors:** Mohammed Al-Dakhil, Walid Ben Romdhane, Salem Alghamdi, Ahmed Abdelrahim Mohamed Ali

**Affiliations:** 1 Advanced Agricultural and Food Technologies Institute, King Abdulaziz City for Science and Technology, Riyadh 11442, Saudi Arabia; 2 Department of Plant Production, College of Food and Agriculture Sciences, King Saud University, Riyadh 11451, Saudi Arabia; walid.brm3@gmail.com (W.B.R.); salem@ksu.edu.sa (S.A.); 3 National Research and Development Center for Sustainable Agriculture (Estidamah), Riyadh 11422, Saudi Arabia

**Keywords:** aquatic plants, salt-stress tolerance, molecular analysis, gene expression, ion homeostasis

## Abstract

Salinity affects the morphological, physiological, and biochemical characteristics of several plant species. The current study was conducted to investigate differential salt tolerance potentials among ten duckweed clones under different salt-stress conditions. Morphological and physiological parameters, including fronds length, fronds number, root length, root number, Na^+^/K^+^, chlorophyll, proline contents, and fresh harvest weight, were recorded for each of the ten duckweed clones collected from different Saudi Arabia regions. Additionally, the expression patterns of seven salt-related genes were monitored in a salt-tolerant duckweed genotype. The results show that the Madinah-2 (*Spirodela polyryiza*) and Al-Qassim (*Landoltia punctata*) clones presented higher performances for all the tested morphological and physiological parameters compared to other genotypes under salt-stress conditions. At concentrations greater than 150 mM NaCl, these aforementioned traits were affected for all the genotypes tested, except Madinah-2 (*S. polyryiza*) and Al-Qassim (*L. punctata*) clones, both of which exhibited high tolerance behavior under high salt conditions (200 mM and 250 mM NaCl). The principal component analysis (PCA) showed that the first five principal components accounted for 94.8% of the total variance among the studied traits. Morphological and physiological traits are the major portions of PC1. Moreover, the expression pattern analysis of *NHX*, *BZIP*, *ST*, and *KTrans* transcript revealed their upregulation in the Al-Qassim clone under salt-stress conditions, suggesting that these genes play a role in this clone’s tolerance to salt-induced stress. Overall, this study indicates that the Al-Qassim clone could be used in a brackish-water duckweed-based treatment program with a simultaneous provision of valuable plant biomass.

## 1. Introduction

Duckweeds represent a family of 36 species of floating aquatic monocytic plants [1,2]. Propagated by vegetative budding, duckweeds are the fastest-growing angiosperms and are spread worldwide [1,3]. Under favorable conditions, duckweeds can cover an entire pond or lake in a few days [4]. Due to their exemplary characteristics (e.g., their fast biomass accumulation, high protein and starch contents, and high rate of nutrient intake), in recent years, duckweeds have drawn scientists’ attention and have been recognized as ideal aquatic plants for use in livestock feed, wastewater treatment, and bio-ethanol production [5,6]. Recently, the adoption of aquatic plants for water management (especially for scantly untreated sources) has significantly increased [7].

Duckweeds have a reduced morphology that is dominated by fronds with few or no roots [8,9]. Natural and anthropogenic mined sources contain the most abundant sodium chloride salt (NaCl). Increases in the abundance of saltwater can cause physiological stress for plants [10]. Salt stress impairs freshwater flora’s development, growth, and tolerance mechanisms in plants [11]. Unlike other angiosperms, duckweeds’ reduced morphology results in a loss of physical barriers between their photosynthetic organs and their nutrient supply [12]. This could explain why duckweeds are particularly sensitive to salt stress, which is a well-known cause of cellular damage and impacts plant growth and development. The physiology and biochemistry of duckweed plants are influenced by salinity [13,14,15]. Saline water is found everywhere; thus, scientists have focused on identifying salt-tolerant genotypes that perform better in saline conditions based on the plants’ morphological and physiological characteristics.

The selection of duckweed genotypes plays a crucial role in improving the use of duckweed for industrial applications [16,17]. Several duckweed species showed the reduced vegetative growth under salt-stress conditions [15,18]. However, resistant clones adopt certain mechanisms that can withstand salinity-induced osmotic tension [19,20], and as a result, an anti-oxidative defense mechanism is activated [18]. These implications have also been observed in the roots [21].

It was also reported that the optimum range for duckweed clone (*Spirodela polyryiza*) growth was recorded between 600 μs/cm and 1400 μs/cm [22]. Elevated levels of salt inhibited the growth, photosynthesis, electron transport, and photosystem II reaction center in *Wolffia arhiza* [23]. However, the characterization of stress-tolerant duckweed genotypes would be needed under changing environmental conditions. The plant adopts complex mechanisms for stress tolerance, leading to a successful adaptation [24,25]. These mechanisms involve stress sensors, stress-signaling molecules, transcription factors, stress-related genes, stress proteins, and efficient scavengers of reactive oxygen species (ROS) [26]. Under salt stress, the duckweed *Spirodela polyryiza* L. accumulated high sodium content, while its potassium and calcium contents were significantly reduced [17]. Interestingly, several osmo-sensing calcium antiporters (OSCA) and potassium inward channels, as well as Na^+^/H^+^ antiporters (SOS1 and NHX), and a Na^+^/Ca^2+^ exchanger, are involved in the response to salt stress [27]. The plant tolerance for abiotic stress such as salinity results from the interaction between the plant genetic source and the environment [28]. The plant genetic sources, genes, are expressed based on biochemical and physiological factors that substantially affect the plant’s ability to tolerate stress [29]. An investigation into the expression of stress-related genes can improve our knowledge of the mechanisms adopted by plants to avoid the negative effects of abiotic stress, as well as the metabolic pathways involved in responding to stressful conditions [30]. The present study was conducted to assess the morphological, biochemical, and molecular differential responses of ten duckweed clones subjected to different salt levels and to select promising genotypes for fodder production under harsh conditions. In the literature, there is little research on recognizing duckweed taxa and species in Saudi Arabia. The current study was designed to evaluate different duckweed clones collected from different locations in Saudi Arabia under different levels of salinity and to conduct a gene expression analysis for the most salt-tolerant duckweed species (*Landoltia punctata*) evaluated under salinity stress.

## 2. Materials and Methods

### 2.1. Plant Materials

Ten duckweed clones were collected from different locations in Saudi Arabia. These duckweed clones had previously been characterized by Al-Dakhil et al. [31] and were deposited in the NCBI database based on their sites of collection as follows: Riyadh (accession OK103562), Dhahran (accession: OK383787), Tohama (accession: OK383788), Al-Baha (accession: OK103563), Jazan (accession: OK383786), Al-Taif (accession: OK383789), Tanomah (accession: OK383790), Al-Qassim (accession: OK247674), Madinah-1 (accession: OK571366), and Madinah-2 (accession: OK247675) [31]. The plants were rinsed with water, cultured, and allowed to multiply under greenhouse conditions at 28 °C and with 14 h of light. The experiment was conducted in a greenhouse facility at the Department of Plant Production, College of Food and Agricultural Science, King Saud University, Riyadh, Saudi Arabia.

### 2.2. Salinity Treatments

Duckweed clones were placed in a hydroponic growing system containing nutrient solution (NS), and the pH of the solution was adjusted to 6.2. The nutrient solution was prepared by mixing the following components in a one-liter vessel: 600 mg of Chem-GroTM fertilizer 8-15-36, 600 mg of calcium nitrate (Ca(NO_3_)_2_), and 373 mg of magnesium sulfate (MgSO_4_) [32]. After the establishment of the duckweed’s plant, the morphological data were computed [33]. For the evaluation of salt tolerance, the duckweed genotypes were transferred into media with different levels of salinity (control, 50 mM, 100 mM, 150 mM, 200 mM, and 250 mM NaCl). After two weeks, plants (fronds) were collected for an ion (Na^+^ and K^+^) content analysis and to determine their biochemical traits (proline, chlorophyll. a, chlorophyll b, and total chlorophyll).

### 2.3. Measurements

#### 2.3.1. Vegetative Growth Parameters

The vegetative traits of the plants were determined, including the frond fresh (FW) and dry weight (DW), the number of fronds (FrN), frond length (FrL), the number of roots (RN), and root length (RL). These traits were determined using standard protocols.

#### 2.3.2. Proline Content

The free proline content was determined using the method of Bates et al. [34]. Proline was extracted from 0.25 g of the fresh fronds of salt-stressed and non-stressed duckweed genotypes. The samples were ground in a mortar with 2 mL of sulfosalicylic acid (3% *w*/*v*) and transferred to a 2 mL tube. They were then centrifuged to pellet the sample tissue. Then, 1 mL of supernatant was transferred to a new tube and reacted with equal volumes of glacial acetic acid and ninhydrin reagent. It was then incubated for 1h in boiling water at 100 °C. The reaction was terminated by placing the reaction tubes in an ice bath. The reaction mixture was vigorously mixed with 2 mL of toluene. After being warmed at 25 °C, the proline content was measured at 520 nm using a spectrophotometer. A standard curve was calculated by measuring (1, 5, 10, 20, 30, 40, and 50 µg/mL) dilutions of the proline stock solution (1 mg/mL).

#### 2.3.3. Ion Content Analysis

Dried samples of the duckweed clones were ground and used to determine the Na^+^ and K^+^ contents. First, 0.15 g of dry material from each duckweed clone was placed into a 50 mL flask. In a fume hood, 5 mL of concentrated sulfuric acid was added to the tube and kept overnight, and 1 mL of 30% peroxide was added. After the oxidation reaction had subsided, the flasks were placed on a heat block for 30 min. After cooling the flasks to room temperature, another 1 mL of peroxide was added before returning the flasks to the digestion block. This step was repeated until the acid in the tube was clear and colorless [35]. The samples were diluted to 100 mL using ddH_2_0 and mixed well. Analyses of the sodium (Na^+^) and potassium (K^+^) contents were carried out via flame atomic absorption (Thermofisher, Waltham, MA, USA, ICE 3000).

#### 2.3.4. Chlorophyll Pigment Contents

A frond sample (0.1 g) was obtained and placed in 10 mL of 80% acetone to determine the chlorophyll a (Chl*a*), chlorophyll b (Chl*b*), and total chlorophyll (Chl*a*+*b*) contents. The absorbance was measured at 645 nm and 663 nm using a spectrophotometer. The chlorophyll was then converted to micrograms per gram of leaf tissue. Chlorophyll a (Chl*a*), chlorophyll b (Chl*b*) content, and total chlorophyll content were calculated according to Arnon [36] using the following equations:

Chl*a* (mg·g^−1^) = [(12.7 × A_663_) − (2.6 × A_645_)] × mL of Acetone _80%_ × sample FW (g)

Chl*b* (mg·g^−1^) = [(22.9 × A_645_) − (4.68 × A_663_)] × mL of Acetone _80%_ × sample FW (g)

Chl*a*+*b* (mg·g^−1^) = [(20.2 × A_663_) − (8.02 × A_645_)] × mL of Acetone _80%_ × sample FW (g)

### 2.4. RNA Isolation and cDNA Synthesis

Duckweed clone (*L. punctata*) samples (100 mg) that had been subjected to normal and salt-stress treatment (150 mM NaCl) were collected at the vegetative growth stage, directly preserved in liquid nitrogen, and then stored in −80 °C freezers. The total RNA was isolated from the fronds using an RNeasy Midi Kit (Qiagen, Valencia, CA, USA) according to the manufacturer’s recommended protocol. The quantity of total RNA was estimated using a Nanodrop 2000 spectrophotometer (Thermo Scientific, Waltham, MA, USA), and the integrity was assessed on a 1.2% denatured formaldehyde agarose gel. The RNA was stored at −80 °C for further experiments. The first-strand cDNA was generated using a Quant script Reverse Transcriptase kit (Qiagen). A mix of 1 µg total RNA, random hexamer primers, and deoxyribonucleotide5-triphosphates (dNTPs) was incubated at 65 °C for 5 min before chilling on ice. The first strand was then reverse transcribed at 42 °C for 1 h using superscript III reverse transcriptase for a final volume of 20 µL. The obtained cDNA was diluted 1:10 (*v*/*v*) with nuclease-free water and stored at −20 °C.

### 2.5. Primer’s Design

Seven salt-related genes were selected to investigate their expression profiles in the salt-tolerant Al-Qassim (*L. punctata*) clone. The primers were designed via the Vector NTI program (Invitrogen, Waltham, MA, USA) using the following features: a primer length of 18–22 bp, Tm (°C) of 58–60, an amplicon product length of 120–200 bp, and C/G 3′ ends. The primers used are listed in Table 1.

### 2.6. Quantitative Real-Time PCR (qPCR)

Quantitative PCR amplification reactions were performed in a final volume of 20 μL containing 50 ng of cDNA, 10 μL of Precision™ 2X qPCR Master mix with sybr green (Biomolecular Technologies, Inc., MO, USA), and 300 nM of each primer. Reactions were conducted in triplicate on a Light Cycler 480 instrument (Roche, Basel, Switzerland), following the manufacturer’s cycling parameters. The thermocycling protocol consisted of 50 °C for 5 min, an initial denaturation at 95 °C for 10 min, 40 cycles each of denaturation at 95 °C for 20 s, 55 °C for 20 s, and 72 °C for 30 s. The expression levels of the selected stress-related genes were monitored on the salt-tolerant Al-Qassim (*L. punctata*) clone at different (control, 24 h, 48 h, 72 h) salt exposure times. The actin gene was used as an internal reference gene. The relative transcript levels of the target genes were calculated using the 2^−ΔΔCT^ method [38].

### 2.7. Statistical Analysis

The data are provided for each experiment as the mean ± the standard error of the mean (S.E.M) of three replicates. Statistical analyses were performed using the statistical package SAS V9.3 (SAS Institute, Raleigh, NC, USA) with a one-way ANOVA. According to the Tukey post hoc test, the mean values marked with different letters on the figures indicate significant differences at *p* < 0.05. Qualitative data were standardized using data transformation techniques to avoid the scaling effect. The XLSTAT statistical software (https://www.xlstat.com/en/ (accessed on 5 September 2023)) was used to perform principal component and hierarchical cluster analysis.

## 3. Results

### 3.1. Effects of Salinity on the Growth and Morphological Features of Duckweeds

**Figure 1:**
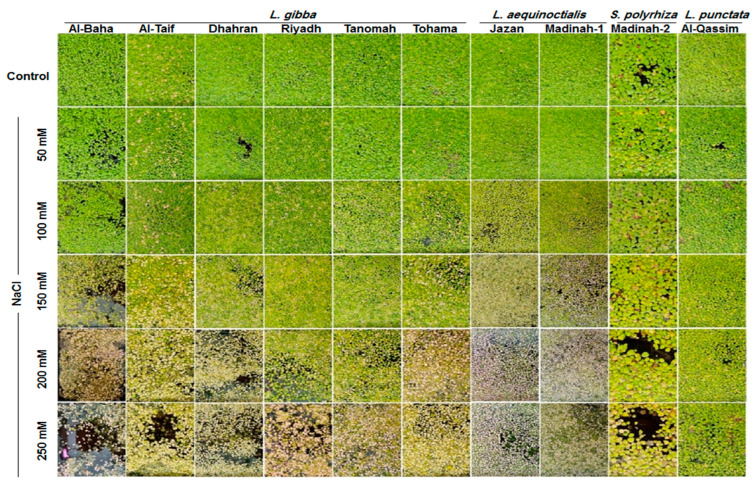
Assessment of salt tolerance of ten duckweed clones collected from different locations in Saudi Arabia under control and different salinity levels.

The morphological features of ten duckweed clones were monitored under normal and different salinity conditions (50 mM, 100 mM, 150 mM, 200 mM, and 250 mM NaCl) to gain insight into the salt tolerance potential of the duckweed clones (Figure 1). The growth of the evaluated duckweed clones was differentially inhibited by the NaCl treatments, and visible damage appeared in the colors of fronds at high concentrations of NaCl, especially at concentrations equal to or above 150 mM NaCl. Interestingly, a green color was revealed on the fronds of the *L. punctate* clone (*Al-Qassim*) grown in 250 mM NaCl (Figure 1).

**Figure 2:**
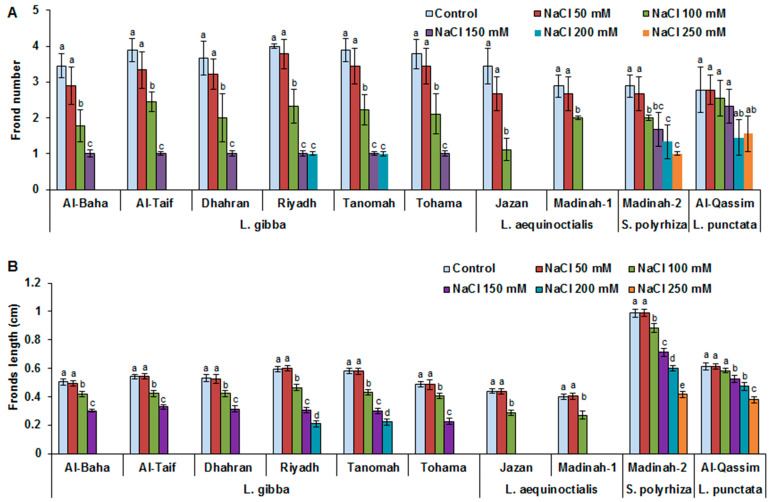
Influence of salinity on duckweed growth and morphological features. Effect of different salt-stress levels on duckweed frond number (**A**). Effect of different salt-stress levels on duckweed frond length (**B**). Values are means ± SD of ten replicates per genotype. Different letters indicate significant differences at *p* < 0.05.

As shown in Figure 2, no significant differences in either frond length (FrL) or frond number (FrN) were recorded under normal conditions or for the 50 mM NaCl treatments among all evaluated duckweed genotypes. By increasing the salinity, the FrN and FrL values were reduced in a dose-dependent manner in all evaluated duckweed genotypes. The reductions in the FrL under 100 mM NaCl compared to that under control conditions were estimated to be 16–25%, 32–35%, 10%, and 4% for the *L. gibba*, *L. aequinoctialis*, *S. polyrhiza*, and *L. punctata* clones, respectively. However, the reductions in FrL and FrN were more pronounced after treatment with ≥150 mM NaCl. The duckweed clones (Al-Baha, Al-Taif, Dhahran, and Tohama) belonging to *L. gibba* tolerated up to 150 mM NaCl (demonstrating mortality at high levels of salt stress), while the genotypes Riyadh and Tanomah tolerated up to 200 mM NaCl. Among the assessed genotypes, the Jazan and Madinah-1 clones (*L. aequinoctialis*) demonstrated the most sensitive behavior to saline stress, surviving only when exposed to NaCl concentrations up to 100 mM. Contrastingly, Al-Qassim (*L. punctata*) followed by Madinah-2 (*S. polyrhiza*) displayed the highest FrN and FrL values under high-salt-stress conditions (250 mM NaCl) (Figure 2A,B).

**Figure 3:**
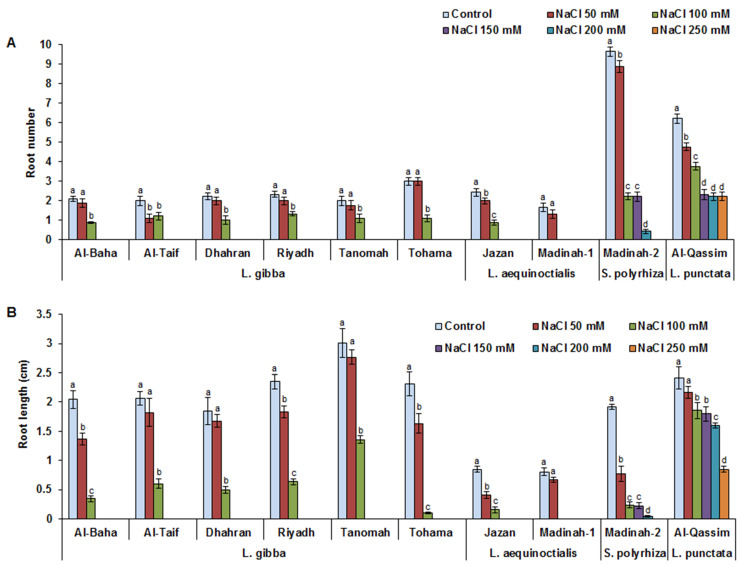
Influence of salinity on duckweed root system. Effect of different salt-stress levels on duckweed root number (**A**). Effect of different salt-stress levels on duckweed root length (**B**). Values are means ± SD of ten replicates per genotype. Different letters indicate significant differences at *p* < 0.05.

The root systems of the duckweed clones were also affected by the salinity, particularly the root number and length, in a dose-dependent manner (Figure 3A,B). This reduction was indiscernible under 50 mM NaCl treatment compared to that of the control conditions; however, the detrimental effects of saline stress were particularly pronounced in plants subjected to treatments greater than or equal to 100 mM NaCl. The reductions in root number under 100 mM NaCl treatment compared to normal conditions were 44–62%, 63–100%, 77%, and 39% for the *L. gibba*, *L. aequinoctialis*, *S. polyrhiza*, and *L. punctata* clones, respectively. The root length was negatively affected in the ranges of 55–95%, 80–100%, 87%, and 23% for the *L. gibba*, *L. aequinoctialis*, *S. polyrhiza*, and *L. punctata* clones, respectively, under 50 mM NaCl treatment compared to that in control conditions (Figure 3B). Interestingly, the Madinah-1 clone showed the greatest root system sensitivity to levels of salinity greater than 50 mM NaCl. However, the Al-Qassim clone’s root system showed the greatest vigor, with a high root number under higher salt-stress conditions (250 mM NaCl) despite 39% and 64% reductions in the root number and length, respectively, under such conditions. All the tested duckweed genotypes demonstrated tolerance up to 150 mM NaCl except for the Jazan and Madinah-1 clones, which showed root sensitivity under high salinity. At high NaCl concentrations (above 150 mM), the Al-Qassim and Madinah-2 clones displayed the highest tolerance.

### 3.2. Effects of Salinity on Duckweed Yield Traits

**Figure 4:**
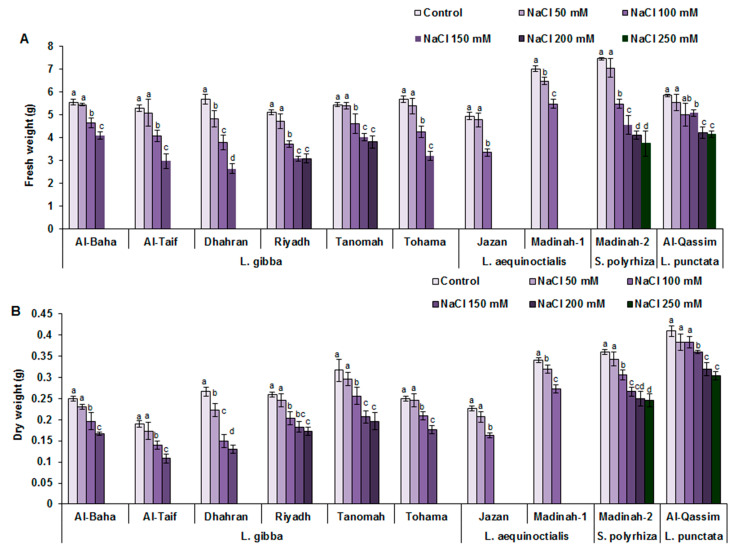
Influence of different levels of salinity on duckweed yield traits. (**A**) Effects of salinity on fresh weight (**A**) and dry weight (**B**) of duckweed genotypes. Values are means ± SD of three replicates per genotype. Different letters indicate significant differences at *p* < 0.05.

Under normal conditions, close fresh weight (FW) values were recorded for the *L. gibba* genotypes, with an average value of 5 g; however, the *L. aequinoctialis* genotypes (Madinah-1 and Jazan) showed significant divergence in their FW values (Figure 4). Among all assessed genotypes, the Madinah-2 clone (*S. polyrhiza*) was found to have the highest FW. For the plants exposed to 50 mM NaCl, Dhahran and Madinah-1 were the only genotypes to show significant decreases in their FW values compared to the control. Compared with those grown under normal conditions, all assessed duckweed genotypes showed decreased FWs at NaCl concentrations of 100 mM NaCl and above, with reductions of 16–34%, 22–32%, 26%, and 14% for *L. gibba*, *L. aequinoctialis*, *S. polyrhiza*, and *L. punctate* genotypes, respectively. At higher NaCl levels, the Jazan and Madinah-1 clones showed no growth at 150 mM, whereas the Riyadh and Tanomah genotypes showed no obvious reductions in their FWs when exposed to 200 mM NaCl compared to those exposed to 150 mM NaCl (Figure 4). Among all the assessed genotypes, the Al-Qassim (*L. punctata*) and Madinah-2 (*S. polyryiza*) clones produced the highest FWs when exposed to 250 mM NaCl compared to the other assessed genotypes.

Compared to normal conditions, salt stress negatively affected the dry weights (DW) of duckweed clones, and the values recorded for all assessed genotypes had a pattern comparable to that of the FW values. Under 100 mM NaCl conditions, Al-Qassim (*L. punctata*) had the highest dry matter content, whereas the Al-Taif clone (*L. gibba*) had the lowest dry matter content. By increasing the NaCl level, a similar downward trend in DW was recorded for all assessed genotypes except for Al-Qassim (*L. punctata*) and Madinah-2 (*Spirodela polyryiza*), which showed tolerance to different levels of NaCl. Compared to the values under normal conditions, the decrease in DW under 200–250 mM NaCl conditions ranged from 30 to 31% and 21 to 26% for Madinah-2 (*S. polyryiza*) and Al-Qassim (*L. punctata*), respectively (Figure 4).

### 3.3. Effect of Salinity on Duckweed Chlorophyll Content

**Figure 5:**
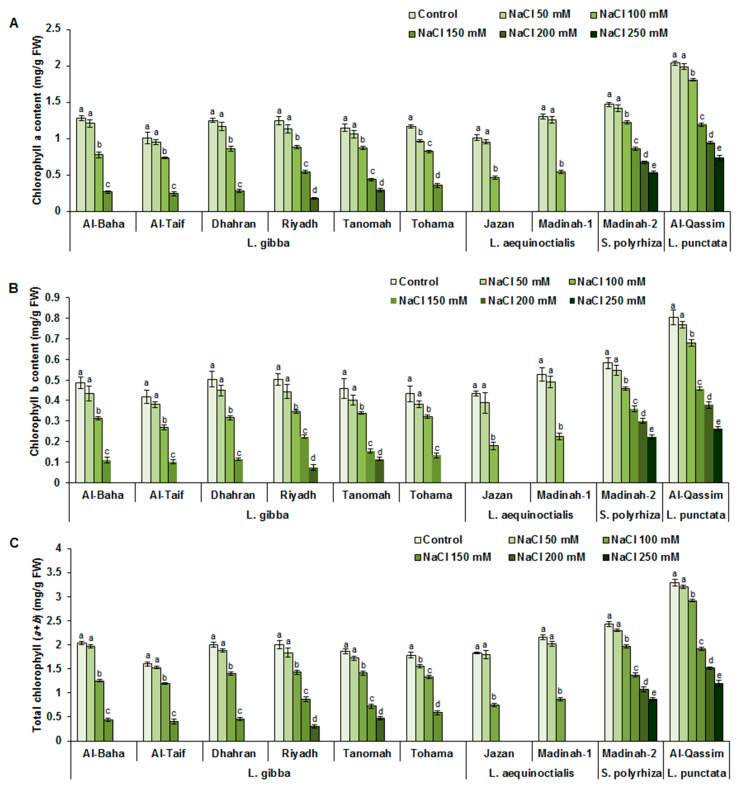
Influence of salinity on duckweed chlorophyll content. Effect of different salt-stress levels on duckweed chlorophyll a content (**A**), chlorophyll b content (**B**), and total chlorophyll content (**C**). Values are means ± SD of three replicates per genotype. Different letters indicate significant differences at *p* < 0.05.

To evaluate the tolerance potential of different duckweed genotypes under normal and salt-stress conditions, their chlorophyll (a, b, and total) contents were determined (Figure 5A–C). With increasing NaCl concentrations, the chlorophyll (a, b, and total) contents of different evaluated duckweed genotypes were reduced in a dose-dependent manner. However, the chl*a*/*b* ratio slightly changed, although no significant differences were observed under the salt-stress treatments (Figure S1). Compared to the plants grown under normal conditions, the chlorophyll (a, b, and total) contents were not significantly affected by the treatment with 50 mM NaCl for any of evaluated duckweed genotypes (Figure 5A–C). In contrast, among the duckweed genotypes exposed to 100 mM NaCl, the Jazan and Madinah-1 clones of *L. aequinoctialis* were characterized by the highest decreases (59%) in chlorophyll (a, b, and total) contents, compared to those recorded under normal conditions, whereas the lowest reductions in chlorophyll contents were recorded for the Madinah-2 and Al-Qassim clones, with values of 43% and 41%, respectively. At high NaCl levels (250 mM NaCl), no growth (death) was recorded for Al-Baha, Al-Taif, Dhahran, Riyadh, Tanomah, Jazan, Madinah-1, and Tohama; however, Al-Qassim (*L. punctata*) and Madinah-2 (*S. polyryiza*) exhibited high tolerance to such conditions, albeit with 64% reductions in their chlorophyll contents compared to those under normal conditions (Figure 5A–C).

### 3.4. Influence of Salinity on Duckweed Na^+^ and K^+^ Contents

**Figure 6:**
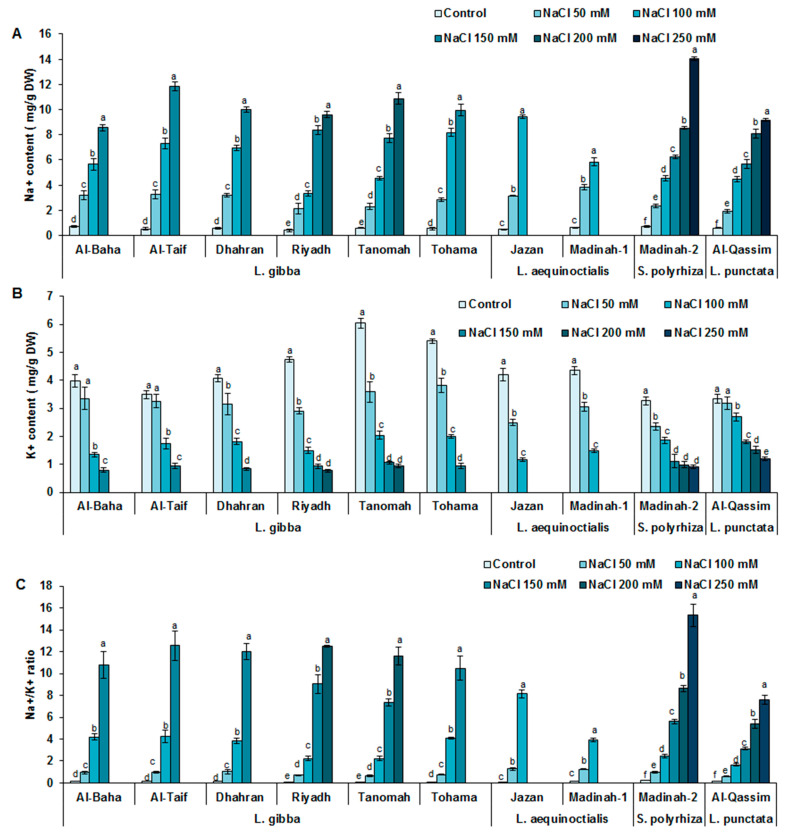
Na^+^ (**A**) and K^+^ ion content (**B**) and Na^+^/K^+^ ratio (**C**) in duckweed subjected to different levels of salinity. Values are expressed as means ± SD of three replicates per genotype. Different letters indicate statistically significant differences according to Duncan’s test (*p* < 0.05).

For all the duckweed genotypes assessed, an increase in Na^+^ content was revealed in response to an increase in salinity. The genotypes Jazan (*L. equinoctials*), Al-Taif (*L. gibba*), Tanomah (*L. gibba*), and Madinah-2 (*S. polyrhiza*) were found to have the highest Na^+^ contents at 100, 150, 200, and 250 mM NaCl, respectively. Notably, Al-Qassim (*L. punctata*) was characterized by the lowest Na^+^ content at all NaCl treatment levels (Figure 6). Contrastingly, all the assessed genotypes showed dose-dependent reductions in their K^+^ contents upon exposure to NaCl. These decreases in K^+^ content ranged from 50% to 68% and from 65% to 72% for the genotypes of *L. gibba* and *L. aequinoctialis*, respectively, and were 43% and 19% for the genotypes of *S. polyrhiza* and *L. punctata*, respectively, under 100 mM NaCl conditions compared to the control conditions (Figure 6).

The high Na^+^/K^+^ ratios exhibited by the Tohama, Tanomah, Al-Baha, and Dhahran genotypes of *L. gibba* and the Jazan and Madinah-1 genotypes of *L. aequinoctialis* were associated with the salt-sensitive behaviors of these genotypes when subjected to salt stress. Contrastingly, compared with the other duckweed genotypes, the Al-Qassim (*L. punctata)* and Madinah-2 (*S. polyryiza*) clones displayed the lowest Na^+^/K^+^ ratios under all NaCl treatments, and these ratios were associated with NaCl-tolerant behaviors. Both *L. aequinoctialis* genotypes (the Jazan and Madinah-1 duckweed clones) showed high degrees of sensitivity to salinity, with plants dying upon exposure to salt concentrations greater than 100 mM NaCl (Figure 6).

### 3.5. Effect of Salinity on the Proline Content of Duckweed

**Figure 7:**
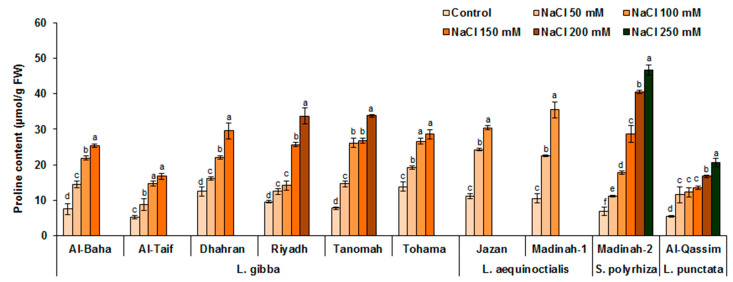
Influence of different levels of salt stress on proline content on duckweed genotypes. Values are means ± SD of three replicates. Different letters indicate significant differences at *p* < 0.05.

In plants, the accumulation of proline in response to salt stress is considered a trait associated with saline tolerance. Consequently, the proline levels were monitored in the evaluated duckweed genotypes subjected to different NaCl treatments. As shown in Figure 7, under 100 mM NaCl treatment, increases of 48–238%, 173–273%, 158%, and 125% were detected in the proline contents of the duckweed species *L. gibba*, *L. aequinoctialis*, *S. polyrhiza*, and *L. punctata*, respectively, compared to their proline contents under normal conditions. Although the Al-Baha, Al-Taif, Dhahran, and Tohama genotypes of *L. gibba*, and the Jazan and Madinah-1 genotypes of *L. aequinoctialis* exhibited similar behaviors in response to salt stress, they had notably differing levels of proline under these conditions, indicating that the accumulation of proline might not be a prominent factor contributing to salt tolerance in duckweeds. Although both genotypes Al-Qassim (*L. punctata*) and Madinah-2 (*S. polyrhiza*) showed high tolerance to salinity (surviving at 250 mM NaCl), they were characterized by completely dissimilar proline content profiles (Figure 7).

### 3.6. Principal Component Analysis for Growth Parameters

**Figure 8:**
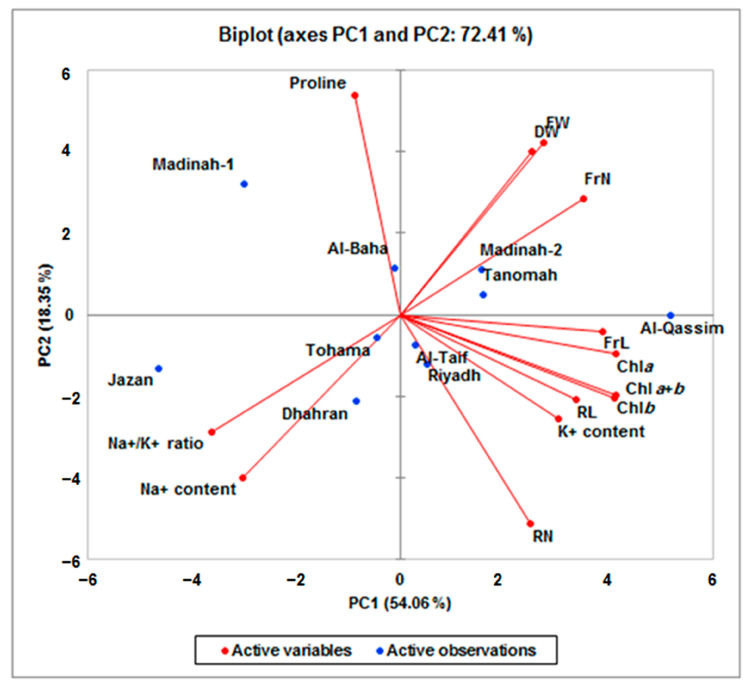
Principal components biplot (PC1 and PC2) of various growth and physiological traits in the ten Saudi dickweed genotypes under salinity-stress conditions (100 mM NaCl). Frond number (FrN), frond length (FrL), fresh weight (FW), dry weight (DW), root number (RN), root length (RL), proline content, chlorophyll a (Chl*a*) content, chlorophyll b (Chl*b*) content, total chlorophyll (Chl*a*+*b*) content, Na^+^ and K^+^ content, and Na^+^/K^+^ ratio.

To identify the primary growth and physiological traits that can be evaluated to assess the salt-tolerance potential of the ten Saudi duckweed genotypes, a principal component analysis (PCA) was accomplished using the average salt tolerance indices (S/C) values for all the evaluated traits under 100 mM NaCl conditions (Figure 8, Table 2).

Both principal components, PC1 and PC2, had eigenvalues greater than 2.3 and contributed to 54.06% and 18.35% of the total variance, respectively (Table 2). The most significant characters (with a high score >0.44) contributing to PC1 were the chlorophyll contents (Chl*a*, Chl*b*, and Chl*a+b*), FrL, FrN, RL, Na^+^/K^+^ ratio, and ion contents (K+ and Na^+^). The proline and Na^+^ contents were the significant variables in PC2 (Figure 8). Concerning the distribution of the duckweed genotypes on the PCA plot, the *L. punctata* genotype (Al-Qassim), which showed the best performance under 100 mM NaCl conditions, was located far from the center of the plot, in the positive patterns of the most growth and physiological evaluated traits, while the *L. gibba* genotypes, especially the Jazan, Tohama, and Dhahran clones, showed poor performances under such conditions in the negative orientation of traits. The genotypes Jazan and Madinah-1 present in PC2 represented the most sensitive genotypes under saline conditions (100 mM NaCl).

### 3.7. Agglomerative Hierarchical Clustering (AHC) Analysis for Salt Tolerance Indices

**Figure 9:**
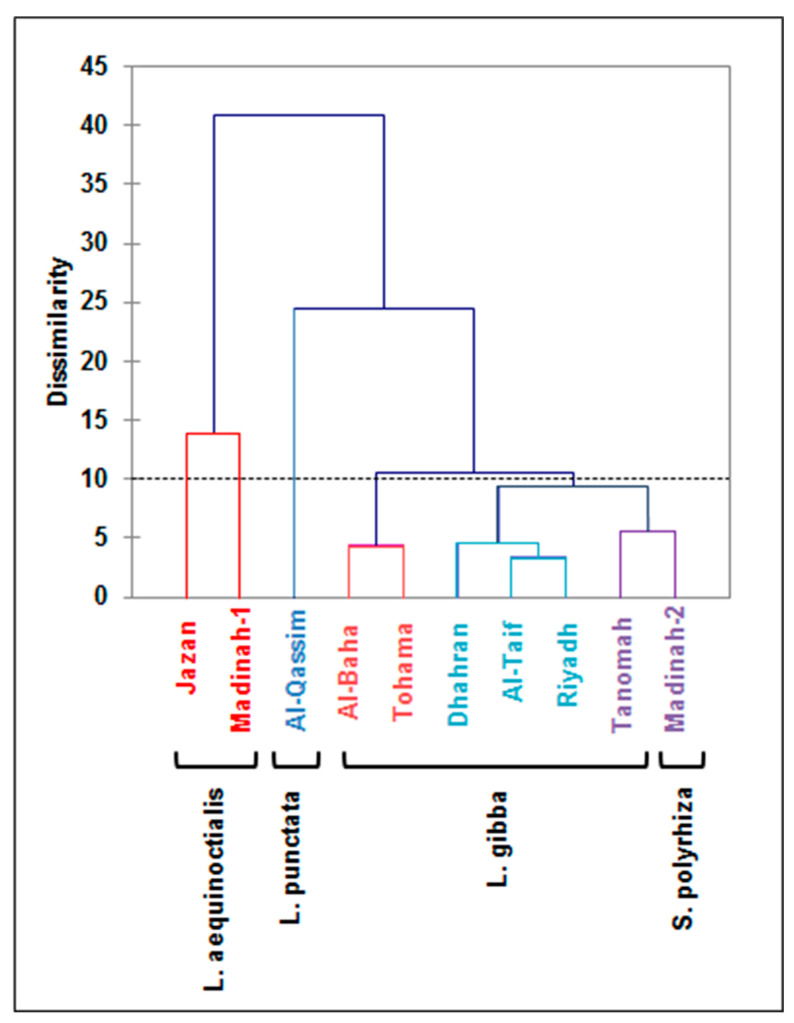
Hierarchical cluster analysis of ten duckweed genotypes from different Saudi Arabia regions for salt tolerance indices based on morphological and physiological traits analyzed under control and 100 mM NaCl conditions. The dendrography was drawn using Ward’s method in XLSTAT software.

The salt tolerance indecies (S/C) for all the morpho-physiological and biochemical traits were used to cluster the ten duckweed genotypes based on their salt tolerance potentials. A hierarchical cluster analysis based on Euclidean distance classified the duckweed genotypes into five clusters (Figure 9). The first cluster regrouped the *L. aequinoctialis* genotypes (Jazan and Madinah-1 clones) and qualified them as highly sensitive to salt stress. However, the Al-Qassim clone (*L. punctata*) was qualified as a highly salt tolerant genotype and constitutes the second cluster. The *L. gibba* genotypes were classified into two clusters with the Al-Baha and Tohama clones qualified as sensitive to salt stress, and the Riyadh, Al-Taif, and Dhahran clones qualified as moderately tolerant genotypes. Finally, the Madinah-2 (*S. polyryiza*) and Tanomah (*L. gibba*) genotypes were regrouped into the fifth cluster and qualified as salt-tolerant genotypes.

### 3.8. Expression Profile of Selected Stress-Related Genes in the Salt-Tolerant Duckweed Genotype

**Figure 10:**
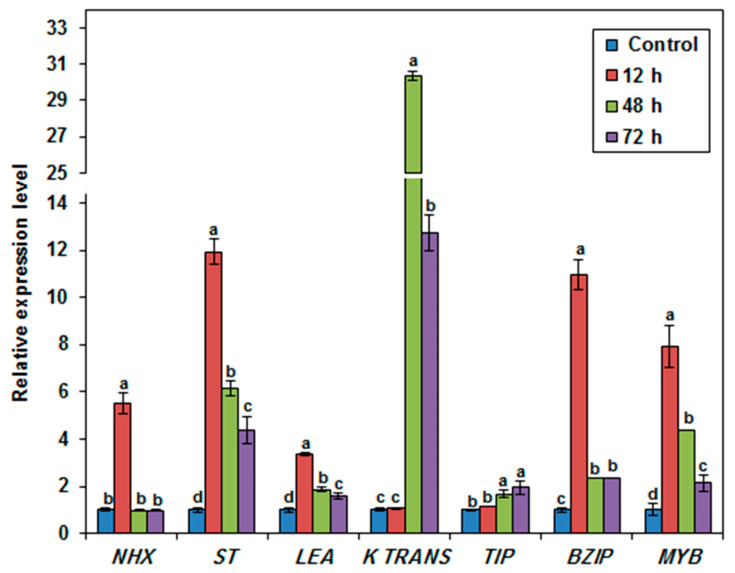
Transcriptional profiles of seven stress-related genes in salt-tolerant Al-Qassim clone (*L. punctata*) under control and sat-stress treatments. Data showed the mean ± SD of three technical replications. Means denoted by the same letter did not differ significantly at *p* < 0.05.

To gain insight into the underlying processes of the salt-stress response adopted by the Al-Qassim clone (*L. punctata*), which exhibited the most tolerant behavior under 250 mM NaCl conditions, expression profiles of seven salt-stress-related genes (NHX, ST, LEA, K Trans, TIP, bZIP, and MYB) were monitored at the vegetative growth stage under normal and salt-stress conditions (150 mM NaCl) at different time intervals (control, 12 h, 48 h, and 72 h) (Figure 10). Expression levels of almost all evaluated stress-related genes were upregulated following 12 h of the salt-stress treatment compared to control conditions, indicating that these genes were involved in plant salt responses. The transcript accumulations of genes involved in the transcriptional activation of salt-stress-response genes, particularly the *bZIP*, stress protein, and *MYB* genes, were upregulated by 11-, 12-, and 8-fold, respectively, relative to the control conditions at 12 h. At the same time (12 h), the expression of the sodium/hydrogen antiporter gene (*NHX*) and the late embryogenesis abundant protein coding gene (*LEA*) were also 5- and 3-fold accumulated, respectively, relative to control conditions (Figure 10). The potassium transporter gene (*K Trans*) involved in K^+^ uptake was induced later and its transcript accumulation reached 30-fold relative to control conditions at 48 h. However, the modification of the transcript accumulations of the aquaporin (*TIP4-1*) gene was less remarkable relative to control conditions and reached only 2-fold after 72 h following NaCl treatment (Figure 10). Overall, the expression patterns of stress-related genes in the Al-Qassim clone (*L. punctata*) indicated that its salt-stress response involved, first, the induction of transcription factors; second, the activation of the sodium/hydrogen antiporter to counter excess sodium; and finally, the high induction of the K^+^ transporter gene in order to enhance K^+^ uptake and establish ion homeostasis.

## 4. Discussion

Duckweeds live in freshwater and occupy lentic habitats [8]. They have attracted increasing interest in recent years due to their promising potential for use in animal food and bioenergy feedstocks, their phytoremediation capacity, and their multipurpose applications [16,39,40]. However, the growth and development of duckweeds are generally affected by salt stress. The primary stresses generated following salt-stress application are osmotic stress and ion toxicity, which cause oxidative stress and lead to reduced growth and photosynthesis [11,41]. The present study investigated the morphological, physiological, and biochemical performances of ten duckweed genotypes under different salt-stress levels. The salt-stress tolerance potential of each duckweed genotype was assessed based on morphological traits (frond length, frond number, root number, root length, fresh weight, and dry weight), biochemical traits (proline contents, chlorophyll contents, and ion (Na^+^ and K^+^) contents). The molecular response was assessed via an evaluation of transcript accumulation of seven salt-stress-related genes in the salt-tolerant genotype. The key approach was to use a multivariate analysis in order to find the performant duckweed genotype under different salinity levels, which could potentially be useful for the production of valuable plant biomass and duckweed-based salted-wastewater treatment programs.

Salinity affects the growth, photosynthesis, mineral nutrients, protein composition, and ionic balance of plants [42,43,44]. The ten evaluated duckweed genotypes displayed differential salt-stress tolerance potentials associated with differential morphological and physiological responses recorded under different salt treatments. By increasing the salinity, almost all the monitored morphological and growth traits, including FrN, FrL, RN, RL, DW, and chlorophyll contents were reduced in a dose-dependent manner in all the duckweed genotypes evaluated. The Jazan and Madinah-1 clones (*L. aequinoctialis*) exhibited the most sensitive behavior to salt stress, which was associated with drastic reductions in their growth traits after treatment with ≥100 mM NaCl. The *L. gibba* evaluated genotypes displayed differential salt-stress tolerance potentials; thereby, Al-Baha, Al-Taif, Dhahran, and Tohama clones exhibited sensitive behavior to NaCl treatment ≥ 150 mM, while the genotypes Riyadh and Tanomah exhibited moderately tolerant behavior to high salt concentrations (200 mM NaCl). These findings suggest the intraspecific phenotypic plasticity of *L. gibba* duckweed. Phenotypic plasticity is advantageous since it could allow the populations to survive and persist across environmental changes [45,46]. Contrastingly, the most salt-tolerant behavior was associated with the Al-Qassim (*L. punctata*) clone, followed by the Madinah-2 (*S. polyrhiza*) clone, for which the highest values of the evaluated traits under high-salt-stress conditions (250 mM NaCl) were recorded. In this regard, the obtained results from *L. aequinoctialis* are similar to those reported by De Morais et al. [15], who mentioned that four evaluated *L. aequinoctialis* clones displayed a sensitive phenotypic behavior characterized by growth inhibition and chlorosis followed by necrosis when subjected to a 50 mM NaCl treatment. Yilmaz [47] revealed the low performance of *L. gibba* duckweed, which was associated with the inhibition of biomass production and root growth rate under increased saline conditions. Several other studies on physiological and biochemical responses of *S. polyrhiza* and *L. punctate* duckweeds under various levels of salinity revealed their tolerance to high salt levels (≥200 mM NaCl) [22,48,49,50].

When the level of salinity increases, Na^+^ accumulation becomes toxic and affects chlorophyll, enzymes, and membranes [11,51]. An increased Na^+^ content was revealed in all evaluated duckweed genotypes in response to increasing salinity. The genotype Jazan (*L. aequinoctialis*) was found to have the highest Na^+^ content at 100 mM NaCl, while Al-Qassim (*L. punctata*), followed by Madinah-2 (*S. polyrhiza*), was characterized by the lowest Na^+^ content under such conditions. The excess Na^+^ accumulation was regretfully associated with a decrease in K^+^ content, which impacts the intracellular Na^+^/K^+^ balance [52]. The high Na^+^/K^+^ ratios exhibited by *L. gibba* (Tohama, Tanomah, Al-Baha, and Dhahran clones) and *L. aequinoctialis* genotypes (Jazan and Madinah-1 clones) were associated with the salt-sensitive behaviors of these genotypes. However, the NaCl-tolerant behaviors of the *L. punctata* and *S. polyryiza* genotypes (Al-Qassim and Madinah-2 clones) were associated with their lower Na^+^/K^+^ ratios under all NaCl treatments. As a result of Na^+^ accumulation, an increase in pigment degradation or altered biosynthesis occurs, resulting in reduced chlorophyll contents that occur in a salt-level-dependent manner [53,54]. Thereby, the present results show that by increasing the NaCl concentration, the chlorophyll contents of the different evaluated duckweed genotypes were reduced in a dose-dependent manner and among the duckweed genotypes exposed to 100 mM NaCl, the sensitive *L. aequinoctialis* genotypes (Jazan and Madinah-1 clones) were characterized by the highest decreases (59%) in chlorophyll contents, whereas the tolerant *L. punctata* (Al-Qassim) and *S. polyryiza* (Madinah-2) genotypes displayed the lowest reductions in chlorophyll contents. These findings suggest that the Al-Qassim (*L. punctata*) and Madinah-2 (*S. polyryiza*) clones involved more efficient Na^+^/K^+^ uptake and translocation mechanisms than that of the other (*L. aequinoctialis* and *L. gibba*) genotypes evaluated.

Several plant species have been found to accumulate proline under salt-stress conditions, and its accumulation has been correlated with stress tolerance [55,56,57]. In the present study, the proline content was increased in all evaluated duckweed clones by increasing salinity. These findings are in line with previous studies, which report that proline is involved in plant responses to environmental stresses, especially in osmotic regulation and ROS removal in order to reduce the deleterious effects of abiotic stress [58,59,60]. Despite their similar behaviors in response to salt stress, the duckweed clones pertaining to *L. gibba* and *L. aequinoctialis* genotypes displayed dissimilar proline contents. In this trend, the salt-tolerant Al-Qassim (*L. punctata*) and Madinah-2 (*S. polyrhiza*) clones exhibited distinct proline content profiles. These findings suggest that the proline accumulation might not be a causal factor for duckweed salt tolerance and the proline content trait may not be a hallmark of salt-stress tolerance potential in duckweed.

The principal component analysis (PCA) offered an efficient description of genetic diversity based on morpho-physiological characteristics under salinity-stress conditions [54,61,62]. In order to classify the evaluated duckweed genotypes based on their salt tolerance potential, a principal component analysis was carried out. Accordingly, the 13 monitored traits were loaded onto PC1 and PC2, and 11 traits were considered efficient screening criteria (with values ≥ 0.44). The distribution analysis of duckweed genotypes on the PCA plot revealed that the *L. punctate* genotype (Al-Qassim), which showed the best performance under 100 mM NaCl condition, was located far from the center of the plot in the positive patterns of the most growth and physiological evaluated traits, while the *L. aequinoctialis* genotypes, especially the Jazan and Madinah-1 clones, showed poor performances under such conditions in the negative orientation of traits. The crucial contribution of chlorophyll content, FrL, and FrN traits supported their uses as important criteria for the screening and selection of the performant duckweed genotypes that can survive under saline conditions. Interestingly, the salt-tolerant genotype Al-Qassim (*L. punctata*), with a potential salt-tolerance ability, was characterized by its capacity to maintain the growth, chlorophyll contents, and intracellular ionic balance under high-NaCl-level conditions. Similarly, Sree et al. [50] reported that among the 34 tested duckweed clones, *L. punctata* clones exhibited a relatively remarkable salt tolerance with a high accumulation of starch. These clones were considered potential feedstock candidates for biofuel production.

To avoid and deal with harsh conditions, plants adopt a complex mechanism for stress tolerance, especially involving stress sensors, stress signaling molecules, transcription factors, stress-related genes, stress proteins, and efficient scavengers of reactive oxygen species [24,25,26]. To decipher the underlying processes of the salt-stress response adopted by the salt-tolerant Al-Qassim (*L. punctata*) clone, the transcript accumulations of seven salt-stress-related genes (*NHX*, *ST*, *LEA*, *K Trans*, *TIP*, *bZIP*, and *MYB*) were monitored under normal and salt-stress conditions (150 mM NaCl) at different time intervals (control, 12 h, 48 h, and 72 h). The present results indicate the upregulation of almost all evaluated stress-related genes after 12 h of the salt-stress treatment when compared to control conditions. Many plant transcription factors including *WRKY*, *bZIP*, *NAC*, *bHLH*, and *MYB* are known to regulate the expression levels of various stress genes and are able to influence the level of salt tolerance in plants [63]. Numerous reports suggest that *NHX*-coding genes play an important role in salt tolerance by promoting the accumulation of Na^+^ into vacuoles [63,64]. Additionally, the mechanisms of K^+^ uptake and translocation in plants under stress conditions are achieved via numerous K^+^ transporters that are crucial for the maintenance of K^+^ homeostasis [52,63]. Several previously published reports demonstrated the enhanced salt tolerance of numerous plant species highly expressing *NHX* genes [65,66,67,68]. The expression profiling of abiotic stress-related genes in response to multiple stresses in rice varieties revealed high induction of *SOS1*, *NHX1*, *HKT-1*, and *SAPK7* in Pokkali and Nonabokra salt-tolerant rice varieties compared to the salt-sensitive IR29 variety [65]. Gálvez et al. [69] confirmed that the wild species *Solanum pimpinelifolium* L, characterized by a high expression level of four *NHX* genes was more tolerant compared to a salt-sensitive cultivated species (*Solanum lycopersicum* L. cv. Volgogradskij). Furthermore, the vacuolar and plasma membrane aquaporin encoding genes are known to play key roles in maintaining water and osmoregulation balance in plants [70]. Consistently, the overexpression of the bamboo aquaporin encoding gene (*TIP4*) confers salinity tolerance in transgenic *Arabidopsis* plants [71]. Taken together and based on an overview of the expression patterns of stress-related genes in the Al-Qassim clone (*L. punctata*), the results suggest that the salt-stress response of the Al-Qassim clone involved the induction of transcription factors first, the sodium/hydrogen antiporter second to counter sodium excess, and finally, a high induction of the K^+^ transporter gene in order to enhance K^+^ uptake and establish ion homeostasis.

## 5. Conclusions

Duckweeds have caught scientists’ attention in recent years due to their exemplary characteristics and have been considered ideal aquatic plants for use in livestock feed, wastewater treatment, and bio-ethanol production. Identification of salt-tolerant duckweed genotypes is highly requested for their use in duckweed-based salted-wastewater treatment programs with the simultaneous provision of valuable plant biomass. In this study, a multivariate analysis was implemented to evaluate the salt-tolerance potentials of ten duckweed clones under different salt-stress conditions. The present findings indicate that the *L. aequinoctialis* genotypes (the Jazan and Madinah-1 clones) exhibited poor performances under all tested saline treatments; however, the *L. punctata* (Al-Qassim) clone, followed by the *S. polyryiza* (Madinah-2) clone presented higher performances for all the tested morphological and physiological parameters compared to the other genotypes under salt-stress conditions. Of all the genotypes evaluated, the Al-Qassim clone may be useful in salted-wastewater duckweed-based treatment programs with a simultaneous provision of valuable plant biomass. Future omics research is necessary to gain an overview of the salt-stress-response mechanisms of the Al-Qassim clone.

## Figures and Tables

**Table 1:** Primer list of salt-stress related genes used in the present study.

Gene Name	Sequence 5′–3′	Description	Reference
NHX2-F	ATGCAGGGTTCCAGGTCAAG	Na^+^/H^+^ antiporter	JZ905382
NHX2-R	AGACCGAATCTGTAGCGGC		
ST-F	AGAGAC TTCGCC TCG ATTGC	Salt-tolerance protein	JZ562398
ST-R	GTTCGTTTGTGGCTCGTTGG		
LEA-F	GAGACACCAGTTTGGGAA CC	LEA	JZ494646
LEA-R	TCTCTGACCACCCCACCTC		
K^+^ TRANSP-F	CAAGAAGGACACGAGAGGG	Potassium transporter	JZ981156
K^+^ TRANSP-R	GACGTGCTTGACGTACATGG		
TIP4-F	GGAACTGGACGGATCACTGG	Aquaporin TIP4-1	JZ546376
TIP4-R	GGCGAACGAAGACTTCAACG		
BZIP-F	CTTTTGCGGATGCAGTGGTC	BZIP2-like protein	JZ983837
BZIP-R	TCATCAGGCTTCACTGTGCC		
MYB306-F	ACCGAGCAGGAGGAGAAGATC	Myb-related protein	JZ982222
MYB306-R	TGCTTGGCCATCTCTATGTC		
ACTIN-F	GGCTACTCCTTCACCACCAC	Beta-actin	[37]
ACTIN-R	GCTCGTAGGTCTTCTCGACG		

**Table 2:** PCA results for the ten duckweed genotypes grown under 100 mM NaCl conditions, Eigenvalues, proportion, and cumulative variance for the first five principal components (PCs) for the salt tolerance of 13 growth and physiological traits.

	PC1	PC2	PC3	PC4	PC5
**Eigenvalue**	7.027	2.386	1.650	0.730	0.532
**Variability (%)**	54.055	18.353	12.691	5.614	4.092
**Cumulative %**	54.055	72.408	85.099	90.713	94.805
**FrL**	**0.743**	0.003	0.129	0.021	0.001
**FrN**	**0.605**	0.136	0.011	0.006	0.182
**RL**	**0.558**	0.073	0.291	0.007	0.060
**RN**	0.305	**0.442**	0.146	0.000	0.038
**Chl*a***	**0.848**	0.016	0.055	0.023	0.043
**Chl*b***	**0.843**	0.066	0.029	0.002	0.019
**Chl*a*+*b***	**0.828**	0.069	0.043	0.003	0.030
**Proline**	0.039	**0.492**	0.289	0.001	0.080
**Na^+^ content**	**0.459**	0.268	0.019	0.147	0.007
**K^+^ content**	**0.453**	0.110	0.383	0.014	0.022
**Na^+^/K^+^ ratio**	**0.659**	0.138	0.052	0.143	0.001
**FW**	0.374	0.303	0.199	0.016	0.030
**DW**	0.312	0.271	0.003	0.349	0.019

Values ≥ 0.44 are presented in bold and indicate important traits for PC. Frond number (FrN), frond length (FrL), fresh weight (FW), dry weight (DW), root number (RN), root length (RL), proline content, chlorophyll a (Chl*a*), chlorophyll b (Chl*b*), total chlorophyll (Chl*a*+*b*), sodium content (Na^+^), potassium content (K^+^), and Na^+^/K^+^ ratio.

## Data Availability

All data is contained within the article or Supplementary Material.

## Supplementary Materials

The following supporting information can be downloaded at: https://www.mdpi.com/article/10.3390/plants12183206/s1. Figure S1: Changes in chlorophyll a/b ratio on duckweed genotypes under different salinity conditions. Values are means ± SD of three replicates per genotype. Different letters indicate significant differences at *p* < 0.05.
