# Supplementary material for: Differential Morpho-Physiological and Biochemical Responses of Duckweed Clones from Saudi Arabia to Salinity
Source: Plants (Basel). 2023 Sep 8;12(18):3206. doi: 10.3390/plants12183206 (PMC10537559; doi:10.3390/plants12183206)
Supplement: Supplementary file 1 [file plants-12-03206-s001.zip › Figure S1.pdf]

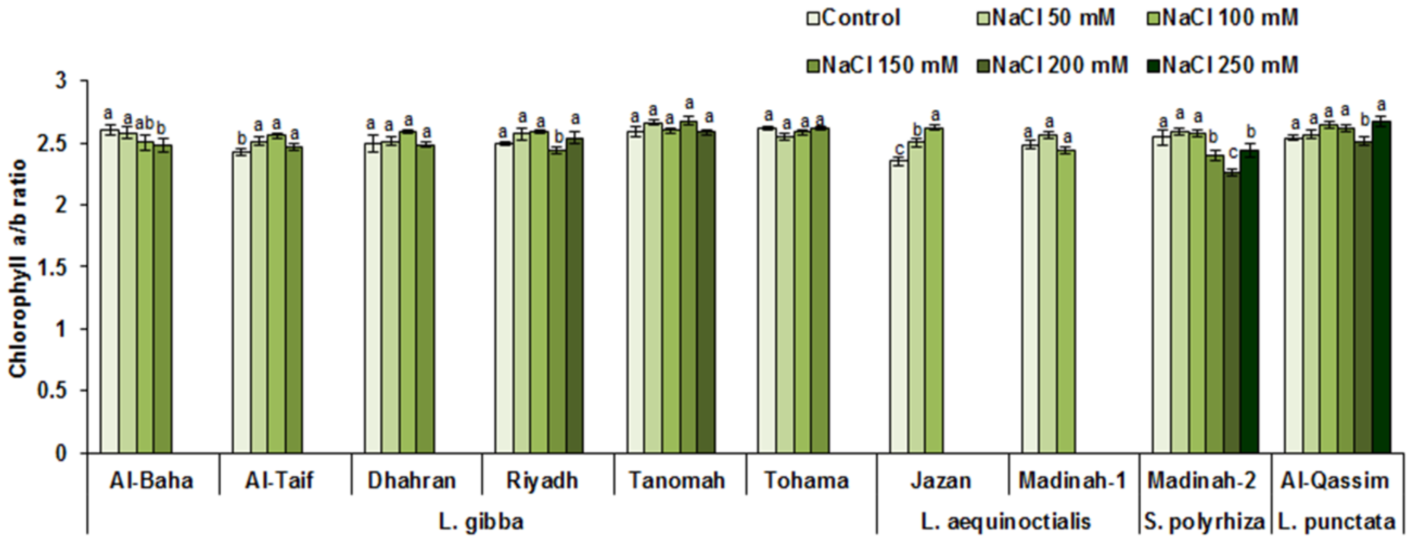


**Figure S1.** Changes in chlorophyll a/b ratio on duckweed genotypes under different salinity conditions. Values are means ± SD of three replicates per genotype. Different letters indicate significant differences at $P < 0.05$.
